# ADHD Symptoms in Middle Childhood: The Role of Child Attachment and Maternal Emotional Availability in an Inpatient Clinical Sample

**DOI:** 10.3390/ejihpe14060104

**Published:** 2024-06-04

**Authors:** Michaela Augustin, Volker Mall, Maria Licata-Dandel

**Affiliations:** 1 Social Pediatrics, TUM School of Medicine and Health, Technical University of Munich, Heiglhofstr. 69, 81377 Munich, Germany; volker.mall@tum.de (V.M.); maria.licata-dandel@charlotte-fresenius-uni.de (M.L.-D.); 2 German Center for Child and Adolescent Health (DZKJ), Partner Site Munich, Heiglhofstr. 69, 81377 Munich, Germany; 3 kbo-Kinderzentrum Munich, Heiglhofstr. 65, 81377 Munich, Germany; 4 Department of Psychology, Charlotte Fresenius University, Infanteriestr. 11a, 80797 Munich, Germany

**Keywords:** child attachment representation, child ADHD symptoms, child inattention, child hyperactivity, emotional availability, interaction quality

## Abstract

Background: Child ADHD symptoms are highly prevalent in middle childhood, alongside impairment in social functioning. The parent–child relationship has been shown to play an important role; however, studies investigating specific facets of the parent–child relationship in ADHD symptomatology in middle childhood have been neglected. We assumed that higher ADHD symptoms were associated with both (1) lower maternal emotional availability (EA) and (2) lower child attachment security. Moreover, (3) we aimed to explore which specific EA dimensions were associated with ADHD symptoms. Methods: In a socio-pediatric clinic in Germany, 71 inpatient mother–child dyads (child age: M = 7.70, SD = 1.06; *n* = 54 boys) were assessed. Clinical data about child ADHD symptoms (Child Behavior Checklist 6–18 subscale “attention deficit/hyperactivity problems”), maternal EA (free play), and child attachment representation (Attachment Story Completion Task, GEV-B) were analyzed cross-sectionally. Results: Controlling for child oppositional behavior and sex, child ADHD symptoms were associated with overall maternal EA, and more specifically non-hostility, but not with child attachment representation. Conclusions: Our results imply that the role of parent–child interaction quality should be considered in the treatment of ADHD. Bidirectional effects cannot be ruled out.

## 1. Introduction

### 1.1. Child ADHD Symptoms

Attention deficit hyperactivity disorder (ADHD) is a neurodevelopmental disorder, beginning in early childhood, which is characterized by symptoms of inattention, hyperactivity, and/or impulsiveness [1]. ADHD symptoms are one of the most prevalent psychiatric symptoms in childhood. Internationally, prevalences rates for child inattention, hyperactivity, or impulsiveness range from 4 to 61% depending on the exact definition of the symptoms, with about 2–7% of children fully meeting the diagnostic criteria for ADHD [2,3,4]. Child ADHD symptoms are associated with impairment in social functioning, such as lower academic achievement [5,6], and are highly comorbid with several other psychiatric symptoms/disorders, such as disruptive behavior problems, conduct disorders, or symptoms of depression [7,8]. Twin studies have demonstrated a high heritability of ADHD (e.g., [9]). However, a child’s environment, and specifically the relationship between the child and their caregiver, has been identified as an important influence factor with regard to the course and progression of ADHD [10,11,12,13].

### 1.2. Parent–Child Interaction Quality

According to attachment theory, the quality of a child`s relationship with their primary caregiver lays the foundation for socio-emotional development [14,15,16]. The link between sensitive caregiving and positive developmental outcomes on the child side, such as the development of emotion regulation skills, is well established [17]. In contrast, negative caregiver–child interactions have been identified as a risk factor of negative developmental pathways. Various studies have demonstrated a link between parent–child interaction quality as concurrent with later child behavior problems, including ADHD [10,18,19,20]. Deater-Deckard [21] argues that the development of self-regulation is affected by parenting practices, such as modelling of appropriate behavior and self-regulation skills. In contrast, insufficient parenting can impair child self-regulation and executive functioning development [22], and thus contribute to the exacerbation of child behavior problems, including ADHD symptoms [23,24]. Research from the Bucharest Early Intervention Project showed that 29% of the children experiencing early disrupted foster care (alongside changes and deficits in the caregiving environment) had ADHD symptoms at the age of 12 years [25]. They found that the elevated ADHD symptoms in children from institutionalized care were mediated by poor child executive functions [26]. In a meta-analysis, Claussen et al. [10] derived that parental sensitivity/warmth was inversely correlated with child inattention and hyperactivity. Furthermore, they found that parental intrusiveness as well as harsh discipline were positively correlated to overall ADHD symptoms. According to coercion theory [27], negative parenting and a child’s problem behavior are likely to reinforce each other in the sense of a vicious cycle, which might also explain associations between parenting and the course of ADHD symptoms. In line with this, Breaux and Harvey [28] found that child ADHD symptoms during preschool predicted less parental global warmth. Another study with school-aged children by Lifford et al. [29] revealed that child ADHD influenced mother–child rejection one year later. However, up to date, none of the existing studies have investigated the link between child ADHD symptoms and interaction quality using the concept of emotional availability (EA). This concept allows us to validly and reliably assess the extent to which a child and their caregiver are able to establish an emotionally connected, authentic, and positive relationship with each other in a dyadic and multidimensional way. On the parent side, sensitivity, structuring, non-intrusiveness, and non-hostility can be assessed, and on the child side, responsiveness and involvement can be rated. Furthermore, the overall connection of the dyad can be assessed using the EA2 clinical screener.

### 1.3. Child Attachment Representation

Attachment theory argues that children develop an attachment representation (i.e., an internal working model of attachment) based on the quality of their relationship experiences with their primary caregivers [14,15,30,31]. While the role of attachment in infancy with regard to child development has been investigated in depth (e.g., [32,33,34]), child attachment beyond infancy has received far less attention. From preschool to primary school age, story stem procedures are a frequently used tool to assess child attachment representation. In this task, children are told to complete stories which activate the attachment system. The stories are codified with regard to coherence and content [35,36,37,38]. Studies have shown that children with ADHD had more insecure and disorganized attachment patterns than children without ADHD [39,40], also see Pallini et al. [41] for a meta-analysis, whereas other studies did not detect such an association [42]. A study using story stems to assess child attachment representation in 4–11-year-olds found that children with ADHD had less secure and more ambivalent and disorganized attachment representations compared to children without ADHD, independent of comorbid oppositional defiant disorder (ODD) and parental education [43]. Another study by Thorell et al. [39] similarly found that attachment disorganization was linked to child ADHD in middle childhood, independent of conduct problems. In contrast, Franke et al. [42] found that differences in attachment representations between children with and without ADHD vanished when controlling for disruptive behavior problems. Thus, findings are still heterogenous in this regard.

### 1.4. The Present Study

Previous studies in this area have shown the following limitations: (1) The vast majority of studies were conducted with children from non-clinical samples, whereas inpatient clinical groups, that are especially vulnerable, have been neglected in research. (2) Most studies investigating the association between parent–child interaction quality and ADHD symptoms have not used multidimensional tools to assess the interaction quality. On the other hand, studies using the EA concept have only been investigated with regard to child externalizing problems more generally, but not specifically with regard to ADHD symptoms. (3) Many studies have used self- or parent-report measures to assess child attachment and/or parent–child interaction quality. Self-report measures have a risk of bias and evoke social desirability [44,45]. (4) Many studies have not controlled for oppositional behavior problems, although oppositional behavior often co-occurs with ADHD symptoms and is related to attachment insecurity [43,46,47] and problems in parent–child interaction quality [48]. Thus, the present study aimed to investigate the link between child ADHD symptoms and child attachment representation, as assessed through an Attachment Story Completion Task [49], and maternal EA, assessed via EA Scales (EAS) [50]. To make sure that this link is independent of child oppositional behavior and to address possible child sex differences, which have been shown to occur in ADHD symptomatology [51,52], we included these variables as control variables. We hypothesized that child ADHD symptoms are related to both (1) lower maternal EA and (2) lower child attachment security. Furthermore, (3) we aimed to explore which dimensions of maternal EA were particularly associated with ADHD symptoms.

## 2. Materials and Methods

### 2.1. Sample and Procedure

**Figure 1:**
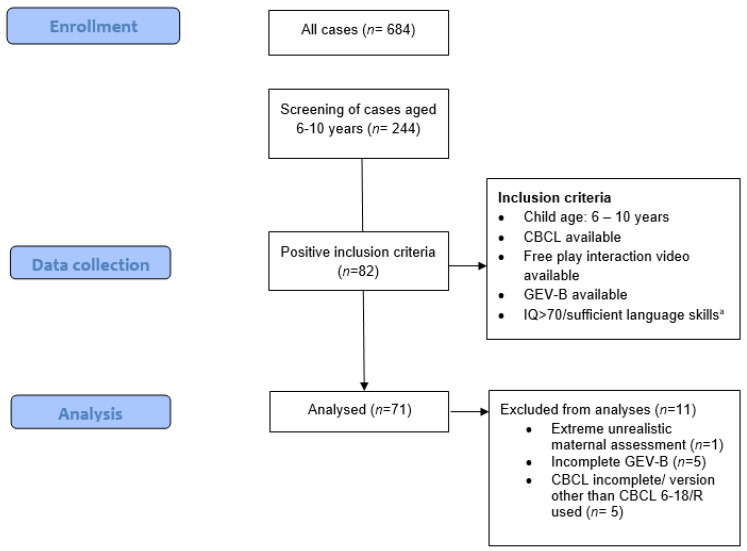
Participant flow chart. ^a^ One child had an IQ = 68 with good enough language skills to perform the Attachment Story Completion Task validly.

Our sample consisted of 71 mother–child dyads who were inpatients in a socio-pediatric clinic in southern Germany between February 2017 and July 2022. During their inpatient stay (average 4 to 6 weeks), all children were evaluated with several diagnostic measurements and received standard care, including individual therapeutic sessions and/or medical support as needed. The data for this study were from these routine care diagnostical procedures, which were usually conducted at the beginning of the inpatient stay, and were retrieved and retrospectively analyzed in accordance with the BayKrG Art. 27. According to BayKrG Art. 27, patient data collected within a regular clinical can be used for research purposes by the hospital. Thus, we screened data from all the inpatients according to the inclusion criteria. We included participants if the children were 6–10 years old; if the measurements for ADHD symptoms, EA, and attachment representation were available as part of their clinical diagnostic procedures; and if their IQ and language skills were good enough to validly perform the Attachment Story Completion Task (IQ > 70). Figure 1 provides an overview of the selected data. All participants were diagnosed by clinical psychologists according to the International Classification of Diseases (ICD-10; [53]) during their inpatient stay (please note: not all children received ADHD full diagnoses, as shown in Table 1). For this study, ADHD symptoms were quantified using the DSM-oriented subscale inattention-hyperactivity of the CBCL/6-18R questionnaire [54], as filled in by the parents. Videotaped free-play interactions were rated by trained staff to assess the mother–child interaction quality according to the concept of the EAS. Child attachment representation was assessed by using a German adaptation of the Attachment Story Completion Task (GEV-B). For both observational measurements, 100% of the videos were rated by one rater (R1) and 35% of the videos were recorded by a second rater (R2) to test for interrater reliability. All raters were certified to codify of the specific measurements.

### 2.2. Measurements

GEV-B [49]: The GEV-B is a German adaptation of the Attachment Story Completion Task [55], which is a projective procedure for middle childhood used to assess children’s attachment representations. A trained staff used figures to act out attachment-relevant everyday situations up to a critical point, and the child was supposed to complete the story and act out the rest of the scene. The instrument included five stories containing different increasingly stressful attachment-relevant themes. The content was codified by a trained rater according to predefined content and structural rules, with an attachment security score being assigned for each story as well as an overall attachment security score (from 0 = extremely insecure, to 4 = very secure). Based on this score, an attachment type could be assigned (secure, insecure–avoidant, insecure–ambivalent, disorganized). In this study, the overall attachment security score was used. A high interrater reliability of was found for the GEV-B in different samples (Cohen’s κ between 0.77 and 0.92) [56]. Convergent validity with the Adult Attachment Interview has been shown [35].

Emotional Availability Scales (EAS, 4th Edition, [50]: The EAS were used to assess the interaction quality between mother and child. The aim of the procedure was to determine the extent to which both interaction partners succeed in creating an authentic, emotionally positive, and well-regulated bond within the relationship. The scales of sensitivity, structuring, non-intrusiveness, and non-hostility referred to the assessment of the mother, and the scales responsiveness and involvement referred to the assessment of the child. All six subscales could each be assessed globally on a 7-point Likert scale (with 7 as the best assessment) or more differentiated by further subscales. For the present study, we focused on the maternal scales, using a sum score across all maternal EA scales (=overall maternal EA) and the global scores for each maternal scale. Furthermore, in the EA2 clinical screener, the overall connection of the dyad was assessed. The EA2 clinical screener consisted of a 100-point scale divided into four zones: emotionally available (81–100), complicated (61–80), detached (41–60), and problematic < 40. Convergent and divergent validity of the procedure with numerous instruments have been found [57].

Child Behavior Checklist (CBCL/6–18R, [54]: This questionnaire contained 120 items to assess behavioral problems, emotional problems, somatic complaints, and social skills. The items could be formed into nine first-order scales: anxious/depressed; withdrawn/depressed; somatic complaints; social problems; thought, sleep, and repetitive problems; attention problems; rule-breaking behavior; aggressive behavior; and other problems. In addition, three second-order scales could be calculated: total problems, internal problems, and external problems. Furthermore, DSM-oriented subscales could be formed: affective symptoms, anxiety symptoms, physical symptoms, inattention-hyperactivity symptoms, oppositional behavior symptoms, and dissocial symptoms. For the present study, the DSM-oriented subscale inattention-hyperactivity symptoms was used as an outcome variable for ADHD symptoms, and the DSM-oriented subscale oppositional behavior was used as a covariate. Internal consistency was good for all scales, with Cronbach’s α > 0.70 (except for the thinking problems scale) [54].

### 2.3. Statistical Analyses

To explore associations between child inattention-hyperactivity, maternal EA, and child attachment representation, bivariate and partial correlations controlled for child sex and child oppositional behavior were conducted. To detect if maternal EA and child attachment were independently associated with ADHD symptoms, a multiple linear regression was conducted, with the maternal EA sum score and the global attachment score as predictors; child sex and the oppositional behavior score as covariates; and with the inattention-hyperactivity score as the outcome variable. The results were based on α = 0.05. Analyses were performed using SPSS statistical software version 28.0 [58].

## 3. Results

### 3.1. Patient Characteristics

After patient data screening (*n* = 244 cases aged 6–10 years), a total of 71 participant’s data were included for analysis (Figure 1). Participant characteristics and descriptives of the study variables can be found in Table 1. Regarding interrater reliability, scores were good to very good for EA: ICC = 0.82, for sensitivity, ICC = 0.96, for structuring, ICC = 0.66, for non-intrusiveness ICC = 0.74, for non-hostility ICC = 0.86, and for the clinical screener. For the global attachment representation score, the interrater reliability was very good: ICC = 0.91.

### 3.2. Maternal Overall EA, Child Attachment Representation, and ADHD Symptoms

Partial correlations controlled for child sex and child oppositional behavior (Table 2) revealed that the maternal EA sum score was correlated to child inattention-hyperactivity, *r_part_* = −0.261, *p* = 0.031. The child attachment representation score was correlated to child inattention-hyperactivity in bivariate correlations, *r* = −0.244, *p* = 0.040; however, the correlation was not significant when controlling for child sex and oppositional behavior (see Table 2), *r* = −0.138, *p* = 0.259. Consistently, a multiple regression model applying both maternal EA and child attachment score as predictors showed that ADHD symptoms were predicted by the maternal EA sum score (*p* = 0.049), but not by child attachment representation (*p* = 0.472), F(4,66) = 17.85, *p* < 0.001, R ^2^_adj_ = 0.49 (Table 3).

### 3.3. Specific Maternal EA Dimensions and ADHD Symptoms

Regarding specific maternal EA dimensions, maternal non-hostility was correlated to inattention-hyperactivity in bivariate, r = −0.281, *p* = 0.017, and partial correlations, r = −0.270, *p* = 0.025 (see Table 2), whereas the other specific dimensions were not correlated to inattention-hyperactivity.

## 4. Discussion

### 4.1. Summary and Discussion of the Results

The present study aimed to evaluate the role of maternal EA, child attachment representation, and ADHD symptoms in middle childhood in inpatient mother–child dyads. We found that maternal EA was negatively correlated with child ADHD symptoms, with the non-hostility subscale being especially negatively associated with ADHD symptoms. Child attachment representation was negatively correlated with child ADHD symptoms only in bivariate correlations, whereas the association vanished after controlling for child sex and oppositional behavior.

In our sample, only 8.5% of the mothers had EA2 clinical screener scores in the emotionally healthy zone. This is in line with previous findings indicating that interaction quality is impaired in dyads with children showing behavior problems, including ADHD [10,18,19,20]. Our finding that overall maternal EA was negatively associated with ADHD symptoms is in line with other studies investigating a combined score for parent–child interaction quality dimensions. For example, Miller et al. [59] found that a combined maternal caregiving behavior score at 9 months was associated with ADHD in middle childhood. Our result supports our hypothesis that insufficient parenting might negatively affect child self-regulation development, which is a risk for ADHD symptoms [21,22,23,24]. On the other hand, taking into account the cross-sectional design used in our study with possible bidirectional effects, our finding might indicate that a child’s ADHD symptoms challenge the caregiver’s overall parenting. This would be in line with the studies by Breaux and Harvey [28] and Lifford et al. [29], who found that child ADHD predicted less parental global warmth and more maternal rejective behavior. Future longitudinal research should be conducted to investigate causal pathways using the concept of EA. The link between parent–child interaction quality and child ADHD is also addressed by psychotherapeutic interventions. For instance, interaction therapy, an intervention that aims to enhance parent–child relationships, has shown to be an effective treatment option for child ADHD by reducing child ADHD symptoms and improving parenting behaviors [60].

Regarding specific facets of maternal EA dimensions, non-hostility turned out to be negatively correlated with ADHD symptoms even after controlling for oppositional behavior. This finding is in line with previous research identifying parental hostility as an important precursor of child ADHD. Romano et al. [61] found that high and persistent trajectories of hyperactivity were predicted by hostile parenting. In a study by Sellers et al. [62], maternal hostility predicted later child ADHD symptoms as well as aggression. According to emotion socialization theory, hostile parenting deprives children of the possibility to learn adaptive self-regulation strategies [41,63]. On the other hand, recent twin and adoption studies also suggest that behaviors consistent with ADHD symptoms in the early years of life (e.g., child impulsivity/activation) may evoke hostile parenting [64,65].

In our study, the lack of significant correlations between child ADHD and other specific EA dimensions, namely maternal sensitivity and non-intrusiveness, is in contrast with findings from Claussen et al. [10]. However, some existing studies indicate that maternal sensitivity/warmth and intrusiveness might not be linked to child ADHD. For example, Kashdan et al. [48] found that ADHD symptoms were neither associated with parental warmth nor maternal intrusiveness (in contrast to paternal intrusiveness) in boys aged 5 to 12 years. Pauli-Pott et al. [66] found that the link between maternal responsiveness/sensitivity and ADHD was completely mediated by child reward-related control capacity at 5 years, and that the association between maternal responsiveness/sensitivity and child ADHD symptoms vanished by controlling for child ODD symptoms. In a study by Keown [67], maternal (in contrast to paternal) sensitivity at age 4 was not related to self- and teacher-rated ADHD symptoms two-and-a-half-years later. Furthermore, they found that maternal intrusiveness was not correlated to maternal ratings of child ADHD and only correlated to teacher-rated inattention, but not hyperactivity-impulsivity. Xing and Wang [68] found that parental hostility, but not warmth, moderated the link between corporal punishment and child externalizing problem behavior. In terms of parental structuring, there are a lack of comparative data investigating if the level of parental structuring is associated with child ADHD symptom severity. On the one hand, it is conceivable that a child’s symptoms could make it difficult for parents to have success with their structuring effort; on the other hand, parents of children with ADHD might show great effort to structure their child’s environment, as structuring/organization is often an important component of parental interventions [69], thus possible associations might not become evident. Future research should be conducted in this regard.

Regarding child attachment representation, as expected, the majority of children had insecure attachment patterns (*n* = 14 for B vs. *n* = 57 for A, C, D). We found that the association between the degree of secure attachment representation and ADHD symptoms vanished when controlling for child sex and oppositional behavior. This result supports findings from other studies focusing on ADHD and attachment at a representational level in middle childhood; Forslund et al. found that disorganized attachment was observed to have effects on ODD symptoms and conduct problems, but not on ADHD in middle childhood [70,71]. In line, Franke et al. [42] showed no independent effect of child attachment on ADHD when controlling for externalizing behavior problems. In contrast, as displayed in a systematic review by Wylock et al. [11], other studies using story stem methods showed significant associations between child attachment and ADHD. Findings are still heterogeneous and might vary depending on several variables such as the used measurement, control variables, and studied population. It is conceivable that specific insecure categorial attachment patterns (such as C, D) might be a better predictor than the global security score; however, due to the small sample sizes of the specific categories, we could not investigate this in our sample. As this is the first study investigating inpatient dyads, results should be replicated in a larger inpatient clinical sample.

In our study employing a multiple linear regression model, maternal EA turned out to be associated with child ADHD symptoms, whereas child attachment representation was not. Some studies indicate that a child’s concurrent relationship experiences might be a better predictor for social functioning than (early) attachment (for an overview, see NICHD Early Child Care Research Network [72]). In their study, investigating more than 1000 children, the relation between early child attachment and behavior problems in middle childhood vanished when entering parenting quality. Moreover, their results suggest that children might respond to changes in parenting quality rather than to absolute levels of parenting. It should be taken into account that acute conditions that necessitated hospitalization in our clinical inpatient sample, often alongside extreme parental burden, might have led to changes in interaction quality over a certain time period. This might be one possible explanation of why the concurrent interaction quality rather than attachment representation was linked to ADHD in our study. Furthermore, a change in interaction quality due to hospitalization would give us an idea of why the child attachment representation was not linked to maternal interaction quality in our sample. Child attachment is assumed to be more stable than interaction quality and might be a result of experiences with maternal, but also other caregiver experiences [72]; thus, the actual maternal interaction quality in the acute situation might not be correlated to a child’s attachment representation. Interestingly, in the study by Dekkers et al. [43], additional explorative analyses within the ADHD group revealed that attachment representation was not linked to parent–child relationship quality. Unfortunately, in this study, we were not able to examine this assumption by repeated measurements or longitudinal data.

### 4.2. Strengths and Limitations

To the best of our knowledge, this is the first study to investigate maternal EA, child ADHD symptoms, and child attachment representation in middle childhood in an inpatient clinical sample. To close a gap in the research, we investigated a very highly vulnerable inpatient sample, focusing mainly on outpatient participants. We have contributed to a better understanding of the associations between mother–child interaction quality, child attachment representation, and ADHD symptoms by using high-quality standardized multidimensional observational measurements codified by certified raters, which have less risk of bias than self-rating measurements. Additionally, we controlled for oppositional behavior, which often co-occurs with our study variables but which several previous studies neglected to control for.

However, our results must be interpreted against the background of some limitations: First, the evaluation of clinical data from inpatient stays did not allow us to investigate longitudinal pathways. Thus, the cross-sectional data did not allow for interpretations of causality. As displayed, it is conceivable that interaction quality might affect ADHD symptoms but, in turn, that the symptoms might negatively affect the interaction quality in terms of a vicious cycle. Larger-scale longitudinal studies are needed to detect causal pathways, addressing interaction quality in the concept of EA and child ADHD. Furthermore, as most of the accompanying inpatient parents were mothers, we were only able to access data from mothers. As indicated by previous research, the results might be different for fathers (e.g., [48,66]). Furthermore, we could not control for maternal mental health outcomes in our study, since standardized parental mental health data were not part of the routinely assessed clinical data in the socio-pediatric clinic. Thus, we cannot rule out the possibility that maternal psychopathology might have affected EA and child symptoms. Additionally, it must be mentioned that ADHD symptoms were rated by the parents. However, the CBCL DSM-oriented scales have been validated with clinical expert ratings, including diagnoses [73,74]. We would like to point out that a high score on the DSM-oriented subscale “inattention/hyperactivity” does not automatically justify a full ADHD diagnosis, even if both are highly correlated. Thus, we can derive from our study that symptom severity was correlated with EA, whereas no statement can be made about the full diagnosis of ADHD. Also, due to the lack of a control group in our study, we could not compare clinical vs. non-clinical children. Moreover, due to the naturalistic clinical setting the data were retrieved from, the children received different individual treatment care. Although the data were mainly assessed at the beginning of their inpatient stay, we cannot exclude the possibility that previous individual or concurrent treatment (e.g., child medication) might have interfered with the assessed outcomes. It would be interesting to investigate disorder- and treatment-specific patterns in a larger sample, using standardized diagnostic interviews such as the Kinder-DIPS [75].

Finally, it must be mentioned that due to the limited sample size, the power was too low to detect all effects with sufficient probability (bivariate correlations: 1-β > 0.99 for large effects, 1-β = 0.74 to 0.99 for medium effects, 1-β = 0.13 to 0.71 for small effects; multiple regression: 1-β > 0.98 for large effects, 1-β = 0.71 to 0.98 for medium effects, 1-β = 0.13 to 0.68 for small effects).

## 5. Conclusions and Practical Implications

The identification of factors associated with child ADHD are essential for prevention and intervention. Our results imply that the role of parent–child interaction quality should be considered in the treatment of ADHD. This may be achieved by parent education, aiming to improve the understanding of the disease, as well as by specifically working on hostile interaction patterns through intervention, e.g., in parent–child interaction therapy. Thus, the vicious circle of negative interactions can be broken at an early stage, which might positively influence the course of ADHD.

## Figures and Tables

**Table 1:** Characteristics.

Characteristics	*n* (%)	M	SD	Range
Maternal age (years)		37.28	6.54	24.00–52.00
Child age (years)		7.70	1.06	5.83–10.42
Child sex				
Male	54 (76.1)			
Female	17 (23.9)			
Child diagnoses (ICD-10) ^a^				
Emotional disorder (F93)	35 (49.3)			
ADHD (F90.0)	30 (42.3)			
Hyperkinetic conduct disorder (F90.1)	8 (11.3)			
Conduct disorder (F91)	22 (31.0)			
Other ^b^	23 (32.4)			
Child CBCL subscale scores				
Inattention/hyperactivity score		69.48	8.71	50.00–80.00
Oppositional behavior score		67.56	8.53	50.00–80.00
Maternal EA				
Overall EA (sum score)		18.12	2.83	11.00–24.50
Sensitivity		3.69	0.82	1.50–6.00
Structuring		4.12	0.96	1.00–6.00
Non-intrusiveness		5.06	0.91	3.50–7.00
Non-hostility		5.25	0.93	3.00–7.00
Maternal EA clinical screener (continuous score)		67.8	12.78	40–100
Maternal EA clinical screener zones				
Emotionally available	6 (8.5)			
Complicated	36 (50.7)			
Detached	27 (38.0)			
Problematic	2 (2.8)			
Child attachment representation				
Global attachment representation score		2.00	0.83	0.40–3.60
Representation classification				
A	16 (22.5)			
B	14 (19.7)			
C	29 (40.8)			
D	12 (16.9)			

^a^ Multiple diagnoses possible. ^b^ Other diagnoses include other behavioral and emotional disorders, with onset usually occurring in childhood and adolescence (F98; *n* = 9); disorders of social functioning, with onset specific to childhood and adolescence (F94; *n* = 5); reaction to severe stress, and adjustment disorders (F43; *n* = 3); eating disorders (F50; *n* = 3); pervasive developmental disorders (F84; *n* = 2), non-organic sleep disorders (F51; *n* = 1); tic disorders (F95; *n* = 1); habit and impulse disorders (F63; *n* = 1); specific developmental disorders of speech and language (F80; *n* = 1).

**Table 2:** Partial ^a^ (and bivariate ^b^) correlations between variables.

	1.	2.	3.	4.	5.	6.	7.
1. Inattention-hyperactivity							
2. Maternal overall EA (sum score)	−0.261 * (−0.187)						
3. Sensitivity	−0.231 (−0.129)	0.889 ** (0.888 **)					
4. Structuring	−0.097 (−0.072)	0.767 ** (0.767 **)	0.681 ** (0.678 **)				
5. Non-intrusiveness	−0.229 (−0.100)	0.663 ** (0.657 **)	0.417 ** (0.419 **)	0.241 * (0.234 *)			
6. Non-hostility	−0.270 * (−0.281 *)	0.837 ** (0.830 **)	0.729 ** (0.714 **)	0.477 ** (0.477 **)	0.435 ** (0.413 **)		
7. EA maternal clinical screener	−0.203 (−0.129)	0.857 ** (0.851 **)	0.948 ** (0.945 **)	0.646 ** (0.635 **)	0.390 ** (0.392 **)	0.739 ** (0.719 **)	
8. Child global attachment score	−0.138 (−0.244 *)	0.208 (0.195)	0.169 (0.151)	0.139 (0.123)	0.270 * (0.246 *)	0.079 (0.094)	0.213 (0.210)

^a^ Partial correlations were controlled for child oppositional behavior and sex. ^b^ Bivariate correlations are reported in brackets. * *p* < 0.05, ** *p* < 0.01.

**Table 3:** Prediction of ADHD symptoms by maternal EA sum score and child attachment representation score.

Predictor	*B*	ß	*SE*	*p*	*CI*
Maternal EA (sum score)	−0.54	−0.18	0.27	0.049 *	−1.08; −0.002
Child attachment representation (global score)	−0.69	−0.07	0.95	0.471	−2.58; 1.21
Child oppositional behavior ^a^	0.69	0.68	0.09	<0.001	0.52; 0.87
Child sex ^a^	−1.21	−0.06	1.76	0.49	−4.71; 2.30

^a^ Child oppositional behavior and sex were entered as covariates. * *p* < 0.05.

## Data Availability

The dataset and the analysis code can be made available on request. We would like to point out that the data can only be provided anonymized, without any person-identifying data.
